# Urban Lifestyle and Climate‐Driven Environmental Exposures: Immunological Consequences for Pediatric Respiratory Allergies

**DOI:** 10.1002/iid3.70248

**Published:** 2025-08-13

**Authors:** Zahra Kanannejad, Walter Robert Taylor, Milad Mohkam, Mohammad Amin Ghatee

**Affiliations:** ^1^ Allergy Research Center Shiraz University of Medical Sciences Shiraz Iran; ^2^ Mahidol Oxford Tropical Medicine Research Unit Bangkok Thailand; ^3^ Centre for Tropical Medicine and Global Health, Nuffield Department of Medicine University of Oxford Oxford UK; ^4^ Professor Alborzi Clinical Microbiology Research Center Shiraz University of Medical Sciences Shiraz Iran; ^5^ Department of Microbiology, School of Medicine Yasuj University of Medical Sciences Yasuj Iran

**Keywords:** climatic change, cytokines, IgE, lifestyle, pediatric respiratory allergy, Th2 cells, urbanization

## Abstract

Pediatric respiratory allergic diseases, including asthma and allergic rhinitis, are increasingly recognized as significant global health concerns, with rising prevalence rates linked to environmental changes driven by urbanization and climate change. This review explores the impact of climatic factors such as temperature fluctuations, shifting precipitation patterns, and dust storms on air pollution and its consequences on respiratory allergic diseases in children. Evidence suggests these environmental exposures increase allergen loads and profoundly influence immune system function. Air pollutants and airborne allergens promote Type 2 helper T‐cell (Th2)‐skewed responses, leading to elevated IgE production, eosinophilic inflammation, and airway hyperreactivity. Additionally, epithelial barrier dysfunction caused by oxidative stress triggers the release of alarmins such as thymic stromal lymphopoietin, interleukin‐33 (IL‐33), and IL‐25, that activate innate lymphoid cells, and amplify allergic sensitization. Long‐term exposure to pollutants also disrupts immune tolerance by impairing regulatory T‐cell (Treg) activity and promoting persistent airway inflammation. This review highlights how these immunological pathways contribute to the severity and chronicity of allergic diseases in pediatric populations, with special attention to studies conducted in regions prone to dust storms. Understanding these mechanisms is critical for developing targeted public health strategies, improving air quality, and mitigating the health impacts of climate change on children.

**Abbreviation list**
AhR: aryl hydrocarbon receptor
CO₂: carbon dioxide
DCs: dendritic cells
DEP: diesel exhaust particles
FEV1: forced expiratory volume in 1 s
IgEi: immunoglobulin E
ILC2: type 2 innate lymphoid cells
IL [e.g., IL‐4, IL‐5, IL‐13, IL‐33]: interleukin
MMPs: matrix metalloproteinases
NADPH: nicotinamide adenine dinucleotide phosphate
NF‐κB: nuclear factor kappa‐light‐chain‐enhancer of activated B cells
NO₂: nitrogen dioxide
O₃: ozone
PAHs: polycyclic aromatic hydrocarbons
PM2.5: particulate matter ≤ 2.5 μm
ROS: reactive oxygen species
SCFAs: short‐chain fatty acids
Th2: T helper 2 cells
TLRs: toll‐like receptors
TNF‐α: tumor necrosis factor‐alpha
Tregs: T regulatory cells
VOCs: volatile organic compounds

## 1 Introduction

Allergic rhinitis and asthma are most common chronic respiratory conditions in children worldwide. The prevalence of allergic rhinitis has increased significantly over the past few decades, affecting approximately 10%–30% of children globally, with higher rates in urbanized regions [1] whilst asthma affects around 8%–12% of children in developed countries, with incidence rates rising due to environmental and lifestyle changes [2].

Urbanization has led to significant shifts in lifestyle, dietary habits, and environmental exposures. The rapid expansion of urban areas has increased pollution levels, reduced biodiversity, and resulted in a greater reliance on processed foods, all contributing to immune system dysregulation. Children growing up in urban settings experience diminished microbial exposure compared to their rural counterparts, leading to impaired immune tolerance and a heightened risk of allergic diseases. The “hygiene hypothesis” and the more recent “biodiversity hypothesis” suggest that reduced early‐life microbial exposures due to modern urban lifestyles contribute to immune system imbalances, favoring allergic sensitization [3, 4]. Additionally, indoor air pollutants, reduced access to green spaces, and increased exposure to synthetic chemicals exacerbate immune imbalances, leading to increased prevalence and severity of pediatric asthma and rhinitis [5].

Climate change is another critical factor influencing pediatric allergic rhinitis and asthma. Rising global temperatures and elevated carbon dioxide (CO_2_) levels have intensified pollen seasons, increasing the duration of airborne pollen and allergenicity [6]. Studies have shown that climate‐induced changes in vegetation lead to increased pollen exposure, exacerbating allergic rhinitis and asthma symptoms in children [7]. Furthermore, extreme weather events, such as wildfires, hurricanes, and flooding, contribute to poor air quality and promote mold proliferation, further triggering allergic reactions [8]. The combination of air pollution and climate‐driven changes in allergen exposure creates a complex interplay that heightens immune system dysfunction in susceptible pediatric populations.

The immune system plays a crucial role in mitigating the effects of urbanization and climate change on allergic diseases. Increased exposure to pollutants and allergens can enhance epithelial‐derived cytokine release, such as thymic stromal lymphopoietin (TSLP), interleukin (IL)‐25, and IL‐33, which drive innate lymphoid cells (ILCs) activation and T helper (Th)‐2 inflammation [9]. Moreover, oxidative stress caused by environmental pollutants can lead to epigenetic modifications that predispose children to allergic rhinitis and asthma [8]. Understanding these mechanisms is essential for developing targeted interventions to mitigate the impact of environmental changes on pediatric allergic diseases.

This review explores the immunological consequences of urbanization and climate change in shaping pediatric asthma and allergic rhinitis. By examining key environmental and immune system interactions, we aim to highlight the urgent need for preventive strategies and public health interventions to mitigate the rising burden of these allergic diseases in children.

## 2 Urbanization and Pediatric Respiratory Allergy Risk

Several studies have investigated the relationship between urbanization and the rising prevalence of pediatric respiratory allergy. Urban environments are characterized by increased exposure to air pollutants, loss of biodiversity, and altered lifestyles, all of which contribute to immune dysregulation and heightened allergic responses in children. Some studies have investigated the relationship between urban settings and the worldwide risk of pediatric asthma and allergic rhinitis (Table 1).

**Table 1:** Effect of urbanization on pediatric respiratory allergic diseases.

Author, year	Location	Study design	Age	Sample size	Type of diseases	Key findings	Reference
C. Cingi., 2005	Eskişehir‐Turkey	Comparative cohort	14–16 years	Urban: 850/Rural: 350	Allergic rhinitis	↑ Incidence of positive skin prick tests among subjects in urban areas compared to rural areas	[10]
Akmatov M.K., 2020	Germany	Retrospective	All age	Urban: 32,400,372/Rural: 14,028,047	Asthma	↑ Risk of asthma associated with living in a densely populated area	[11]
Levin M.E., 2020	South Africa	Cohort	12–36 months	Urban: 1,185/Rural: 398	Asthma, Allergic rhinitis	↑ Risk of asthma in urban communities	[12]
Norbäck D., 2018	China	Cohort	3–6 years	Urban: 29,262/Rural: 1520	Asthma, allergic rhinitis	↓ Asthma and rhinitis in children in suburban or rural areas	[13]
Dostál M., 2014	Czech Republic	Cohort	0–10 years	Urban: 466/Rural: 455	Allergic rhinitis	↓ Allergic rhinitis in rural areas due to less air pollution	[14]
Stoner A.M., 2013	USA	Cohort	5.5 years	Urban: 5,800/Rural: 1,100	Asthma	↑ Risk of asthma hospitalization associated with urbanization	[15]
Valet R.S., 2011	USA	Cohort	0–5.5 years	Urban: 52,168/Rural: 38,317	Asthma	↓ Asthma emergency department visits in children living in a rural area	[16]
Midodzi W.K., 2010	Canada	Cohort	0–2 years	Urban: 5,823/Rural: 2,602	Asthma	↑ Asthma development in rural central metropolitan areas	[17]
Midodzi W.K., 2007	Canada	Observational	0–11 years	Urban: 10,945/Rural: 2,570	Asthma	↓ Risk of asthma in children from a farming environment compared with children from rural non‐farming environments	[18]
Bråbäck L., 2004	Sweden	Comparative	17–20 years	Urban:1,119,437/Rural: 197,548	Asthma, rhinitis	↑ Asthma in farming and non‐farming environments due to environmental changes	[19]
Shima M., 2003	Japan	Cohort	6–9 years	Urban: 1,020/Rural: 838	Asthma	↑ Asthma in children living near significant trunk roads	[20]
Alicia Guillien., 2024	Europe	Cohort	8.1 ± 1.6 years	Total:1,033	Asthma, rhinitis	↑ Risk of asthma and rhinitis in Children from exposure to NO2 and road traffic	[21]
Wrightson S., 2025	New Zealand	Retrospective	Not specified	Total:6,134	Asthma	↑ Asthma in children living in densely populated areas.	[22]
Kanannejad Z., 2023	Iran	Retrospective	1–18	Total:211	Asthma	↑ Risk of childhood asthma hospitalization in an urban setting	[23]

### 2.1 Urbanization and Air Pollution

**Figure 1:**
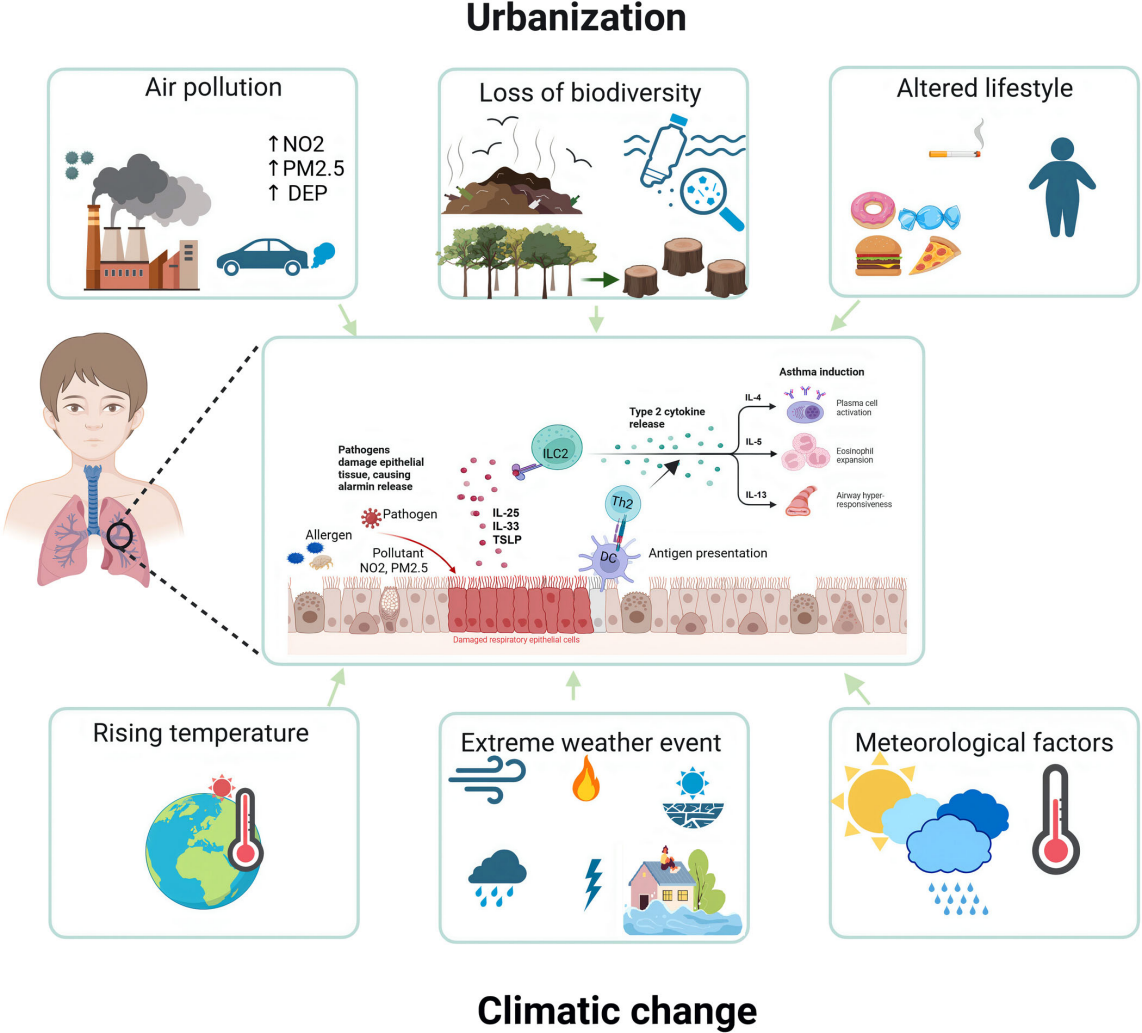
The interplay between urbanization and climate change contributes to increased asthma incidence through environmental and lifestyle factors. Air pollution, biodiversity loss, and altered lifestyles, along with rising temperatures, extreme weather events, and meteorological changes, trigger immune system responses that lead to airway inflammation and asthma development. Pollutants and allergens damage respiratory epithelial cells, activating innate immune pathways (ILC2 and alarmins) and adaptive immune responses (dendritic cells and Th2 cytokines). This results in eosinophilic inflammation, mucus overproduction, and airway hyperresponsiveness, driving chronic asthma progression.

In most industrialized countries, people living in urban areas are generally more susceptible to allergic respiratory conditions than those in rural areas. A large cohort study by Gauderman et al. (2015) found that children living in urban areas with high levels of nitrogen dioxide (NO_2_) and delicate particulate matter 2.5 (PM2.5) had significantly lower lung function and a higher incidence of asthma compared to those in less polluted areas [24]. Road traffic is the primary source of air pollution in most urban areas, and research has shown that living close to high‐traffic roads is linked to reduced respiratory health [25]. Air pollution has been linked to asthma exacerbation, including more bronchial hyper‐responsiveness, increased medication use, and a higher rate of emergency department visits and hospitalizations (Table 1). The effect of air pollutants on lung function varies based on several factors, including the specific type and concentration of the pollutant in the environment, the duration of exposure, the total ventilation of exposed individuals, and the interaction between air pollution and airborne allergens like pollen and fungal spores [26]. Air pollutants affect respiratory allergic diseases differently, including interaction with pollen grains, inducing inflammation, and modulating immune responses (Table 2; Figure 1).

**Table 2:** Impact of urbanization and air pollution on immune responses in the respiratory allergic disease.

Immune component	Role in allergic responses	Effect of urbanization & air pollution	Reference
Epithelial barrier (Airway epithelium)	Prevents allergen entry and maintains lung homeostasis	PM2.5, NO₂, and ozone disrupt tight junctions, increasing permeability to allergens	[27, 28, 29]
Alarmins (TSLP, IL‐33, IL‐25)	Initiate Th2 immune responses via dendritic cells	Increased secretion due to oxidative stress from pollutants, amplifying allergic inflammation	[30, 31, 32]
Dendritic Cells (DCs)	Present allergens to naïve T cells, driving Th2 polarization	DEP and PAHs enhance DC activation, leading to exaggerated allergic responses	[33]
Th2 Cells	Mediate allergic inflammation via IL‐4, IL‐5, IL‐13	Skewed Th2 polarization due to air pollutants, increasing eosinophilic inflammation	[34, 35]
Regulatory T Cells (Tregs)	Maintain immune tolerance, prevent excessive inflammation	Impaired function due to pollution‐induced oxidative stress, reducing immune regulation	[36]
Eosinophils	Contribute to airway inflammation and hyperreactivity	Increased recruitment due to NO₂ and PM exposure, worsening inflammation	[37]
Mast Cells	Release histamine and leukotrienes upon	Higher activation due to oxidative stress from pollutants	[38, 39]
IgE Antibodies	Mediate allergic sensitization	Elevated by PAHs, NO₂, and DEP, increasing atopic risk	[40]
Neutrophils	Involved in inflammation and airway remodeling	Traffic‐related pollutants induce neutrophilic airway inflammation	[41]
Pro‐inflammatory Cytokines (IL‐6, IL‐8, TNF‐α)	Promote chronic airway inflammation	Upregulated by urban air pollution, contributing to severe allergic responses	[42]
IL‐17, IL‐22 (Th17‐related cytokines)	Contribute to neutrophilic inflammation and airway remodeling	Elevated in response to air pollution, contributing to severe asthma phenotypes	[43, 44]

PM, particulate matter; NO₂, nitrogen dioxide; DEP, diesel exhaust particles; PAHs, polycyclic aromatic hydrocarbons; IL, interleukin; Th, T helper; TNF, tumor necrosis factor; TSLP, thymic stromal lymphopoietin; DCs, dendritic cells; Tregs, regulatory T cells; ROS, reactive oxygen species; IgE, immunoglobulin E.

Air pollution can modify the antigenicity of pollen grains through various physicochemical interactions, leading to increased allergen release and heightened immune responses in sensitized individuals. Pollutants oxidize and degrade the outer pollen shell (exine), exposing and modifying allergenic proteins such as Bet v 1 (from birch pollen) or Phl p 5 (from grass pollen), making them more potent in triggering allergic responses [45]. In addition, diesel exhaust particles (DEP) and PM2.5 can break pollen grains into smaller, respirable particles that penetrate deeper into the respiratory tract, increasing their potential to trigger asthma and allergic rhinitis [46]. Studies reported that children exposed to high pollen counts and elevated PM2.5 levels had significantly higher rates of allergic rhinitis than those in low‐pollution environments [47]. Pollution has also been found to work as an adjuvant for pollen to enhance the severity of immune responses. DEP, for example, promotes the production of cytokines such as IL‐4, IL‐5, and IL‐13, which drive Th2‐mediated allergic inflammation in response to pollen grains [48].

Airborne pollutants contribute to oxidative stress by directly generating reactive oxygen species (ROS) or through biological pathways. These pollutants can generate ROS by activating nicotinamide adenine dinucleotide phosphate (NADPH) oxidase and mitochondrial dysfunction in airway epithelial cells. When ROS levels exceed the capacity of antioxidant defenses such as glutathione, catalase, and superoxide dismutase, oxidative stress occurs, leading to cellular damage. Studies have shown that exposure to DEPs significantly increases oxidative stress markers in airway epithelial cells, promoting inflammation [49]. Similarly, Fan et al. (2022) demonstrated that PM2.5 exposure enhances mitochondrial ROS production, exacerbating lung inflammation [50].

Oxidative stress caused by pollutants leads to lipid peroxidation, protein oxidation, and DNA damage, which disrupts the integrity of airway epithelial cells. The oxidation of cellular components results in the degradation of cell membranes and structural damage, reducing cellular function. Additionally, pollutants impair tight junction proteins such as occludin and claudin, increasing airway permeability and making the lungs more vulnerable to allergens and pathogens. Research has shown that PM2.5 exposure induces oxidative DNA damage in bronchial epithelial cells, leading to apoptosis and inflammation [51]. Moreover, Wu et al. (2021) found that urban air pollution weakens epithelial tight junctions, facilitating the entry of allergens and microbes into the lungs and exacerbating airway diseases. As airway epithelial cells become damaged, they release pro‐inflammatory cytokines that drive further inflammation. Key cytokines involved in this process include IL‐6, which promotes neutrophil recruitment; IL‐8, which attracts neutrophils to the lungs; and tumor necrosis factor (TNF)‐α, which induces further ROS production and perpetuates inflammation. The release of these cytokines is mediated through the activation of NF‐κB and MAPK signaling pathways, which are triggered by oxidative stress. Zeng et al. (2022) demonstrated that urban PM2.5 exposure significantly increased IL‐6 and IL‐8 levels in lung epithelial cells, contributing to chronic inflammation [52]. Evidence showed that oxidative stress‐induced NF‐κB activation in epithelial cells led to sustained TNF‐α release, exacerbating airway inflammation [53]. ILC2 can act as an environmental sensor and significantly contribute to protease allergen‐induced lung inflammation. Studies suggest that inhaled acrylamide may worsen allergen‐induced eosinophilic inflammation in the airways, potentially by affecting the proliferative activity of ILC2 [54].

Air pollution also affects immunity by shifting the balance of Th cell responses. Pollutants, particularly DEP and PM2.5, have been shown to favor a Th2‐dominant immune response, which increases IgE production and the risk of allergic diseases like asthma and allergic rhinitis [55]. This change is marked by increased levels of IL‐4, IL‐5, and IL‐13, which contribute to enhanced eosinophilic inflammation and mucus production in the airways. On the other hand, exposure to pollutants can also promote Th17 responses, which contribute to chronic inflammatory diseases by increasing the production of IL‐17 and IL‐22, driving neutrophilic inflammation and tissue damage [56]. Interestingly, air pollution has been shown to suppress T regulatory (Treg) cells, which are crucial in maintaining immune tolerance and preventing autoimmune diseases. A reduction in Foxp3^+^ Treg cells due to pollution exposure leads to excessive immune activation, increasing the risk of allergic diseases [57].

Beyond the well‐established IgE‐mediated mechanisms, recent evidence highlights the importance of non‐IgE‐mediated hypersensitivity responses, particularly in environmentally driven allergic phenotypes. These responses often involve T cell‐mediated inflammation, oxidative stress, and innate immune activation. Non‐IgE immunoreactivity detectable through leukocyte adherence inhibition testing underscores the relevance of alternative immune pathways in allergic diseases not captured by traditional IgE‐focused diagnostics [58, 59]. Furthermore, recent research emphasizes the importance of endotyping allergic diseases, classifying them based on underlying immunological mechanisms rather than relying solely on clinical symptoms. This framework enables the distinction between eosinophilic and neutrophilic inflammation, IgE‐dependent and non‐IgE mechanisms, and varying cytokine profiles (e.g., Th2 vs. Th17 dominance), which may be differentially influenced by climate stressors such as air pollution, ozone, and dust storms. Recognizing this heterogeneity is crucial for understanding how climate change and pollutants influence distinct allergic disease trajectories, and it supports the need for precision‐based strategies in allergy prevention and treatment.

Airborne pollutants also influence B cell function and antibody production. Several studies have shown that exposure to PM2.5 and polycyclic aromatic hydrocarbons (PAHs) can enhance IgE production, which is associated with allergic diseases and asthma [60]. Additionally, some studies suggest that air pollution may impair the production of protective antibodies, such as IgG, reducing immune defense against infections [61].

### 2.2 Urbanization and Loss of Biodiversity

Urbanization‐driven biodiversity loss affects immune system regulation, contributing to an increased prevalence of allergic diseases and immune dysregulation. The hygiene hypothesis later expanded into the biodiversity hypothesis, suggests that reduced microbial diversity in urban environments alters immune system development, leading to an imbalance between immune tolerance and allergic inflammation [62]. The immune system, particularly in early childhood, requires exposure to diverse environmental microbiota to develop appropriate regulatory mechanisms that prevent excessive immune reactions to harmless antigens. In highly urbanized settings, limited contact with beneficial microorganisms leads to immune dysfunction characterized by a skewed Th cell balance, disrupted Treg activity, and heightened inflammatory responses. One of the key immunological consequences of biodiversity loss in urban areas is the dysregulation of Th1 and Th2 immune responses. In natural environments, frequent microbial exposures promote Th1‐biased responses, which help control excessive Th2 mediated allergic inflammation. However, urban children who experience reduced microbial diversity are more likely to develop Th2‐dominant immune responses, leading to increased production of interleukin IL‐4, IL‐5, and IL‐13, which drive IgE production and eosinophilic inflammation [63]. This shift increases children′s susceptibility to allergic diseases such as asthma and allergic rhinitis. Additionally, reducing microbial exposure impairs Treg function, which is crucial in immune tolerance. Tregs suppress inappropriate immune activation and prevent allergic inflammation; however, their activity is diminished in urban populations with limited exposure to biodiversity. The gut microbiota, another critical component of immune regulation, is also significantly impacted by urbanization and biodiversity loss. Early‐life microbial exposure through diet, environment, and maternal microbiota shapes immune responses and promotes immune tolerance. A study comparing urban and rural populations in Kazakhstan found significant differences in gut microbiome diversity and composition. Urban residents showed decreased microbial diversity, a higher Firmicutes‐to‐Bacteroidetes ratio, and an increased prevalence of specific inflammation‐linked genera, such as Coprococcus and Parasutterella [64]. In contrast, rural inhabitants had greater microbial diversity and a higher abundance of genera linked to anti‐inflammatory effects, suggesting that urbanization may negatively impact gut microbiota and immune health [64].

### 2.3 Urbanization and Altered Lifestyle

Urbanization has also led to significant lifestyle modifications, including diet and physical activity changes, which further contribute to immune dysregulation and allergic diseases. Traditional diets rich in fiber, antioxidants, and omega‐3 fatty acids have been increasingly replaced by highly processed, calorie‐dense foods high in sugar, saturated fats, and additives in urban living. This Westernized diet contributes to immune dysfunction through several mechanisms. First, a lack of dietary fiber reduces the production of short‐chain fatty acids (SCFAs) such as butyrate and acetate, which play a crucial role in maintaining gut barrier integrity and modulating immune responses. SCFAs support the development of Tregs, which are essential for immune tolerance and the prevention of allergic inflammation [65, 66]. A deficiency in SCFA production due to low fiber intake can lead to an exaggerated Th2 immune response, increasing the risk of asthma and allergic rhinitis. Second, diets rich in processed foods and unhealthy fats contribute to systemic inflammation and oxidative stress, further exacerbating allergic conditions [67]. High omega‐6 fatty acids, commonly found in urban diets, promote the synthesis of pro‐inflammatory eicosanoids, which have been linked to airway inflammation and increased asthma severity [68]. Conversely, omega‐3 fatty acids, found in fish and certain plant oils, have been shown to exert anti‐inflammatory effects and improve lung function in children with asthma [69]. Urbanization has led to declining physical activity levels among children, further influencing immune system function and allergic disease risk. Reduced outdoor play due to limited access to green spaces, concerns over air pollution, and increased screen time have contributed to a more sedentary lifestyle [70]. Regular physical activity has been shown to have immunomodulatory effects, reducing airway inflammation and enhancing lung function [71]. Exercise promotes the release of anti‐inflammatory cytokines such as IL‐10 and helps regulate immune responses by increasing Treg activity and reducing Th2‐driven allergic inflammation [72]. A sedentary lifestyle, on the other hand, is associated with increased levels of systemic inflammation, obesity‐related immune dysregulation, and heightened allergic responses. Obesity, which is more prevalent in urban settings due to dietary changes and reduced physical activity, further compounds respiratory allergy risk. Adipose tissue produces pro‐inflammatory cytokines such as IL‐1β, IL‐6, and TNF‐α, contributing to airway inflammation and asthma exacerbation [73]. In addition, adipose tissue secretes bioactive molecules known as adipokines, including leptin and adiponectin. The differential expression of these molecules in obesity may be linked to airway inflammation and asthma. Leptin, a 167‐amino acid polypeptide primarily produced by adipose tissue, plays a pro‐inflammatory role in the immune system [74]. It affects a range of immune cells, including monocytes, macrophages, neutrophils, dendritic cells, NK cells, and lymphocytes, by promoting pro‐inflammatory phenotypes and increasing cytokine release, chemotaxis, ROS production, cytotoxic activity, and phagocytosis [75]. Leptin enhances NK cell activity and stimulates the release of IL‐6 and TNF‐α. It also drives CD4^+^ T cell differentiation toward a Th1 and Th17 profile while suppressing Th2 cytokines and Treg expansion. In obesity, elevated leptin levels contribute to chronic inflammation [76]. In contrast, adiponectin, produced and secreted in large amounts by adipose tissue, is widely recognized for its antidiabetic, anti‐inflammatory, antiatherogenic, and cardioprotective effects [77]. Its molecular mechanisms involve directly affecting inflammatory cells by decreasing ROS, increasing the production of the anti‐inflammatory cytokine IL‐10, blocking the NF‐κB signaling pathway, and reducing inflammatory responses that involve TNF‐α [77]. Notably, adiponectin expression and serum levels are reduced in individuals with obesity, which may contribute to impaired respiratory health [78, 79, 80]. Visceral inflammation, accompanied by an increase in pro‐inflammatory macrophages (M1), is also observed in obese individuals with asthma and may contribute to systemic inflammation and asthma severity [81]. In obese individuals, oxidative stress, cell necrosis products, and an excess of free fatty acids promote the polarization of macrophages towards the M1 phenotype, while reducing the presence of anti‐inflammatory M2 macrophages [82]. Research has shown that obese children are at a higher risk of developing severe asthma and tend to have worse responses to asthma treatments compared to their nonobese peers [83].

## 3 Climatic Changes and Pediatric Respiratory Allergy Risk

The rising global temperatures, higher levels of air pollution, and extreme weather events contribute to the severity and frequency of allergic diseases like allergic rhinitis and asthma in children. These climate factors interact with the immune system, affecting allergic sensitization, immune imbalance, and airway inflammation (Table 3; Figure 1). Understanding the immunological mechanisms involved provides crucial insights into the rising burden of pediatric allergic diseases in the context of climate change.

**Table 3:** Impact of climate changes on immune responses in respiratory allergies.

Climatic factor	Immune component	Effect on allergic responses	Reference
Rising temperatures & prolonged pollen seasons	Th2 cells	Increased activation due to prolonged pollen exposure leads to higher IL‐4, IL‐5, and IL‐13 production, promoting IgE‐mediated allergic responses	[84]
	IgE antibodies	Elevated due to increased pollen exposure and allergenicity, intensifying allergic sensitization	[85]
	Eosinophils	Increased recruitment in response to higher pollen levels, leading to airway inflammation and hyperreactivity	[2]
Extreme weather events (thunderstorms, wildfires, floods)	Mast cell	More frequent degranulation due to exposure to thunderstorm‐generated pollen fragments and wildfire smoke, releasing histamine and leukotrienes	[86]
	Pro‐inflammatory Cytokines (IL‐6, IL‐8, TNF‐α)	Wildfire smoke and extreme weather increase oxidative stress, enhancing the production of inflammatory cytokines	[87]
	Neutrophils	Increased activation by wildfire smoke exposure, worsening airway inflammation, and asthma severity	[88]
Flooding & humidity Cchanges	Epithelial barrier (airway epithelium)	Increased permeability due to mold proliferation, facilitating allergen penetration	[88]
	Innate immune receptors (TLR‐2, TLR‐4)	Mold exposure triggers TLR activation, amplifying airway inflammation and allergic sensitization	[89]
Dust storms & increased airborne particulates	Dendritic cells (DCs)	Enhanced antigen presentation from dust storm particles and pollutants, leading to increased Th2 priming	[90]
	Regulatory T cells (Tregs)	Impaired function due to silica and particulate exposure, reducing immune tolerance and increasing inflammation	[91]
Climate change and air pollution interactions	Oxidative stress pathways	Increased ROS production weakens immune defenses, disrupting immune homeostasis	[92]
	Th17 cells	Elevated responses contribute to chronic neutrophilic inflammation and airway remodeling	[93]

Abbreviations: DCs: dendritic cells; IL: interleukine; Th: T helper; TNF: tumor necrosis factor; TLR: toll like receptor; Tregs: regulatory T Cells.

### 3.1 Rising Temperatures and Prolonged Pollen Seasons

Global warming leads to increased temperatures, directly influencing the concentration and distribution of airborne allergens such as pollen. Warmer climates result in earlier onset, prolonged duration, and increased intensity of pollen seasons, leading to higher pollen loads in the atmosphere [94]. Higher temperatures accelerate plant phenology, leading to earlier flowering and extended pollen release periods [95]. In a multi‐decade analysis of pollen trends across North America, researchers found that pollen seasons started earlier and lasted longer in response to climate warming. For example, ragweed (*Ambrosia artemisiifolia*), one of the most potent allergenic plants, now releases pollen for 20–27 days in some regions compared to previous decades [96]. Similar trends have been observed in European cities, where earlier and prolonged pollen exposure has increased allergic disease prevalence [46]. Rising atmospheric CO₂ levels and higher temperatures enhance photosynthesis, increasing plant growth and pollen production. Experimental studies have demonstrated that plants grown under elevated CO₂ conditions produce significantly more pollen than those grown under current atmospheric conditions [97]. Not only do plants produce more pollen in a warming climate, but the allergenic potential of pollen itself is also increasing. Studies on birch pollen (*Betula spp*.) and grass pollen (*Poaceae*) have demonstrated that pollen grains produced under heat stress contain higher levels of allergenic proteins such as Bet v 1 and Phl p 5, respectively, which intensify allergic reactions [98]. Warmer temperatures have also enabled allergenic plants to expand their geographic range, leading to new pollen sources in previously unaffected regions. For instance, ragweed, historically limited to North America, has spread across Europe due to rising temperatures and changing precipitation patterns [99]. This geographic expansion has introduced novel allergens to populations that previously had no exposure, leading to new cases of allergic sensitization and an increased burden of respiratory allergic diseases.

From an immunological perspective, exposure to high pollen concentrations triggers the activation of dendritic cells in the respiratory mucosa, promoting the differentiation of naive T cells into Th2 cells. This shift enhances the production of cytokines such as IL‐5, IL‐4, and IL‐13, which facilitate B‐cell class switching to IgE production, leading to allergic sensitization and eosinophilic inflammation [100].

### 3.2 Extreme Weather Events

Due to climate change, extreme weather events, including storms, hurricanes, heat waves, wildfires, and heavy rainfall, have become more frequent and intense. These events significantly impact respiratory allergic diseases by altering airborne allergen exposure, increasing air pollution levels, and triggering immune dysregulation. Children are more vulnerable to allergic sensitization and asthma exacerbation due to developing their immune and respiratory systems.

#### 3.2.1 Thunderstorms and Thunderstorm Asthma

Thunderstorms have been linked to severe asthma outbreaks, commonly referred to as thunderstorm asthma. Epidemiological studies have shown that thunderstorm asthma primarily occurs during pollen seasons. The connection between thunderstorm asthma and pollen exposure is supported by consistent epidemiological data, as well as temporal and dose–response relationships. However, experimental data and specific criteria remain limited. It is believed that thunderstorms can concentrate pollen grains near the ground and trigger the release of allergenic particles in respirable sizes when they absorb water and rupture due to osmotic shock. As a result, high atmospheric levels of inhalable allergen‐laden fine particles can quickly enter the lower respiratory tract, triggering an inflammatory response. This theory is supported by a study indicating that higher humidity conditions increase the availability of allergens in the air. Thunderstorms expose individuals to a combination of high electric charges, heavy rainfall, increased humidity, and temperature drops, but the impact of these factors on pollen remains unclear or inconsistent. A well‐documented example occurred in Melbourne, Australia, in 2016, when a massive thunderstorm asthma event led to thousands of emergency department visits and multiple fatalities due to pollen‐induced respiratory distress [101]. This event highlights the severe consequences of climate‐induced extreme weather on respiratory allergic diseases.

Thunderstorm‐generated pollen fragments induce an exaggerated Th2‐mediated response, increasing IgE antibodies, eosinophils, and mast cell activation [102]. Increased release of cytokines like IL‐5, IL‐13, and IL‐4 worsens airway inflammation, which plays a significant role in triggering asthma attacks [103].

#### 3.2.2 Wildfires

Wildfires have increased in both frequency and intensity over the past few decades, and this trend is expected to continue with further climate warming [104]. Wildfires produce enormous amounts of airborne pollutants, including PM2.5, CO, nitric oxide (NO), and volatile organic compounds (VOCs), exacerbating immune responses and allergic diseases. Research has shown that exposure to wildfire smoke during the third trimester of pregnancy and the first 12 weeks after birth can have long‐term effects on respiratory health in children [104]. A study of the 2003 southern California wildfires found a link between PM exposure during these events and acute respiratory symptoms in children, with a notable rise in emergency department visits for childhood asthma [105]. Another study on pediatric asthma patients at National Jewish Health found that exposure to wildfire PM2.5 was linked to lower forced expiratory volume in 1 s (FEV1) among children aged 12‐21 [106].

Exposure to wildfire smoke and its immunotoxic components, including PM, VOCs, and PAHs, has been shown to cause significant alterations in key immune pathways in both animals and humans. These effects can persist for several days to weeks. Specifically, wildfire smoke exposure has been linked to the activation of the aryl hydrocarbon receptor, Toll‐like receptor (TLR) pathways, and NF‐ĸB signaling, all of which contribute to elevated levels of pro‐inflammatory cytokines and reactive oxygen species [107, 108]. Additionally, firefighters exposed to wildfires experience increased pulmonary and systemic inflammation. Serum samples taken 12 h after exposure reveal higher levels of IL‐6 and IL‐12, alongside a decrease in IL‐10 [109, 110]. VOCs and PAHs, organic compounds found in wildfire smoke, have both immunosuppressive and pro‐inflammatory effects [111]. They are metabolized to generate reactive intermediates that bind to DNA, leading to oxidative damage, mutagenesis, and immune cell apoptosis or dysregulation [112]. VOCs, such as formaldehyde and benzene, are known to impair immune cell function, including suppressing lymphocyte proliferation and antibody production [113], while exposure to PAHs can also cause thymus atrophy, as persistent activation of the aryl hydrocarbon receptor (AhR) can impact thymocyte proliferation, ultimately reducing T lymphocyte populations in the thymus [114].

#### 3.2.3 Floods

Heavy rainfall and flooding increase indoor humidity, leading to mold proliferation and heightened exposure to fungal allergens such as Aspergillus and Cladosporium. Damp environments favor the growth of mold and exacerbate allergic respiratory diseases, particularly in children with asthma. A study conducted in the USA found that floods caused by hurricanes may lead to higher concentrations of atmospheric fungal spores [115]. Increased moisture levels and the growth of fungi in flooded homes have also been observed in the months following hurricanes [116, 117]. The rise in atmospheric fungal spore counts was associated with a notable increase in asthma‐related hospital admissions and emergency department visits. After Hurricane Katrina (2005), cases of mold‐induced asthma rose significantly among children in affected areas due to prolonged exposure to mold‐contaminated indoor environments [118].

Mold spores activate pattern recognition receptors (PRRs) such as TLR‐2 and TLR‐4, inducing a vigorous inflammatory response. These receptors recognize fungal components like β‐glucans, chitin, and mannans, leading to the activation of NF‐κB and the release of pro‐inflammatory cytokines, including TNF‐α and IL‐1β [119]. This inflammatory cascade contributes to airway remodeling, chronic inflammation, and increased asthma severity. In addition, certain molds, particularly *Stachybotrys chartarum* (black mold), produce mycotoxins that have immunotoxic effects. Mycotoxins such as ochratoxin A and trichothecenes can suppress immune cell proliferation, impair antigen presentation, and induce apoptosis in lymphocytes [120]. This immunosuppressive effect increases susceptibility to respiratory infections and can exacerbate underlying allergic conditions. Chronic mold exposure may alter the composition of the airway and gut microbiomes, contributing to immune dysregulation. Reduced microbial diversity has increased Th2 polarization and allergic disease progression. Studies have shown that children exposed to high mold levels have lower levels of protective commensal bacteria, leading to increased susceptibility to allergic sensitization and asthma [121].

#### 3.2.4 Dust Storm

Dust storms are extreme meteorological events that transport large amounts of airborne particles, microorganisms, pollen, and pollutants across vast distances, which enhance allergic reactions within atopic asthmatics. During the 2009 dust storm in Sydney, Australia, emergency asthma cases were notably higher in dust storm‐affected areas compared to unaffected regions [122]. Countries frequently affected by dust storms, such as those in the Middle East, bear a significantly higher burden of respiratory allergies, with children being mainly at risk due to their still‐developing immune systems. In Kuwait, an age‐stratified time‐series analysis revealed that children aged 0–14 were the only group significantly affected by dust storms in terms of increased asthma admissions [123]. Additionally, research by Karanasiou et al. (2012) found that children exposed to frequent dust storms had higher rates of allergic rhinitis and bronchial hyperresponsiveness [124].

A 10‐year time‐series analysis of respiratory and cardiovascular morbidity in Nicosia, Cyprus, showed that dust exposure induces the release of pro‐inflammatory cytokines such as IL‐6, TNF‐α, and IL‐1β, contributing to airway inflammation [125]. Silica (SiO₂), primarily originating from minerals such as feldspar and quartz, is the predominant mineral component of Asian sand dust. Prolonged exposure to crystalline silica can lead to silicosis, a chronic occupational lung disease marked by persistent inflammation and fibrosis of lung tissue [126]. SiO₂ activates various immune cells, including macrophages, neutrophils, mast cells (MCs), DCs, T cells, and B cells. These immune cells play a crucial role in regulating the processes associated with silicosis and pulmonary fibrosis through multiple molecular pathways. Both innate and adaptive immune responses contribute to the inflammatory response triggered by silica exposure [127]. When silica particles enter the respiratory tract, APCs, such as DCs, process and present silica antigens to naive T cells. CD4^+^ T cell subsets, including Th1, Th2, Th17, and Tregs, have been shown to contribute to the development of silica‐induced pulmonary fibrosis [128]. In silicosis, an imbalance among Th cell subsets contributes to disease progression [129]. During the inflammatory phase, Th1 cells dominate and help suppress fibrosis via IFN‐γ and IL‐12. As the disease progresses, a shift toward Th2 dominance promotes fibrosis through the actions of IL‐4, IL‐5, and IL‐13 [129]. Th17 cells also play a key role in driving lung inflammation and fibrosis through IL‐17, IL‐22, and IL‐1β, which enhance pro‐inflammatory cytokines and Matrix metalloproteinases (MMPs) [130]. Tregs have stage‐dependent roles: they protect against early inflammation but may exacerbate fibrosis later by promoting TGF‐β, collagen production, and Th17 differentiation while also shifting Th1/Th2 balance toward a Th2 profile [131]. Desert dust contains a variety of both pathogenic and non‐pathogenic bacteria that can lead to infections when inhaled. Among the microbial components, lipopolysaccharide (LPS), a glycolipid found in the outer membrane of gram‐negative bacteria, is known to trigger neutrophilic inflammation in the lungs [132].

### 3.3 Climate Change and Air Pollution: Shifting Patterns

Climate change intensifies the severity and frequency of air pollution events, which in turn indirectly impacts allergic respiratory diseases. Higher temperatures and increased solar radiation accelerate photochemical reactions, leading to a higher production of ground‐level ozone (O₃) [133]. Ozone exposure leads to oxidative stress, airway inflammation, and increased bronchial hyper‐responsiveness in asthmatic children. A time‐stratified case‐crossover study found that exposure to ambient O₃ was associated with an increased risk of emergency department visits for asthma in California [134]. Moreover, extreme heat worsens air stagnation, trapping pollutants such as fine PM2.5, increasing their inhalation and adverse respiratory effects. High humidity can increase the solubility and deposition of airborne allergens like pollen and fungal spores, enhancing allergic reactions. Conversely, low humidity contributes to the suspension of airborne particulate matter, worsening air pollution‐related allergic diseases. Additionally, humidity influences indoor allergen levels, fostering mold and dust mite proliferation and exacerbating allergic rhinitis and asthma. A study in Tehran found that increased PM2.5 levels, influenced by temperature and humidity fluctuations, were associated with higher asthma hospitalizations [134]. Strong winds can disperse pollutants, reduce local concentrations, or transport airborne allergens and pollutants over long distances. In regions affected by dust storms, such as Iran and the Middle East, wind‐driven particulate matter carries allergens, heavy metals, and microbes, triggering immune responses that worsen asthma and allergic rhinitis [135]. Rainfall can temporarily cleanse the air by removing particulate pollutants and allergens. However, shifting precipitation patterns due to climate change can lead to prolonged droughts, increasing dust storm frequency, and allergen exposure [136]. Additionally, post‐rainfall spikes in pollen and mold levels can trigger allergic reactions, particularly in children with heightened immune sensitivity [84].

## 4 Implications for Public Health and Future Research

The increasing prevalence of pediatric respiratory allergic diseases, driven by urbanization and climate change, presents a significant public health challenge. Addressing this issue requires a multidisciplinary approach that includes public health policies, urban planning, climate adaptation strategies, and further immunological research.

From a public health perspective, efforts should focus on reducing children′s exposure to air pollutants and allergens through improved air quality monitoring, stricter emissions regulations, and urban greening initiatives. Expanding green spaces, promoting biodiversity, and implementing indoor air quality improvements can help mitigate the adverse effects of urbanization on immune system development. Additionally, public awareness campaigns emphasizing the impact of climate change on respiratory health are essential to drive policy changes and community action.

Future research should aim to elucidate further the immunological mechanisms underlying environmental influences on allergic diseases. Studies exploring the epigenetic effects of pollutants, the role of microbiome diversity in immune tolerance, and the long‐term impacts of early‐life exposure to allergens are crucial. Investigating personalized interventions, such as microbiome‐targeted therapies and precision medicine approaches, could offer innovative disease prevention and management solutions.

As climate change continues to alter environmental determinants of health, interdisciplinary collaboration between researchers, policymakers, and healthcare providers is vital to develop adaptive strategies that protect vulnerable pediatric populations from the growing burden of respiratory allergic diseases.

## 5 Conclusion

The complex interplay between urbanization, climate change, and pediatric respiratory allergic diseases highlights the urgent need for a comprehensive understanding of environmental influences on children′s health. As the prevalence of asthma and allergic rhinitis continues to rise, particularly in urban areas, it is clear that climatic factors such as rising temperatures, shifting precipitation patterns, and increasing air pollution play a significant role in shaping immune system responses and allergic sensitization. The evidence suggests that these environmental changes contribute to immune dysregulation, promoting Th2‐skewed inflammation, oxidative stress, and epithelial barrier dysfunction, all of which exacerbate allergic diseases in children. By recognizing these environmental determinants and their immunological consequences, we can work toward more effective public health strategies, policy interventions, and clinical management approaches. Mitigating the impact of climate change on pediatric respiratory allergies requires reducing air pollution, improving urban air quality, and fostering greater environmental awareness and resilience within communities.

## Author Contributions

**Zahra Kanannejad:** conceptualization, investigation, writing – original draft, writing – review and editing, supervision. **Walter Robert Taylor:** writing – original draft, writing – review and editing. **Milad Mohkam:** writing – original draft. **Mohammad Amin Ghatee:** writing – original draft.

## Ethics Statement

The authors have nothing to report.

## Consent

The authors have nothing to report.

## Conflicts of Interest

The authors declare no conflicts of interest.

## Data Availability

Data sharing not applicable to this article as no datasets were generated or analyzed during the current study.
